# Supplementary material for: A 3D morpho-space of sepal geometry reveals the importance of organ curvature
Source: Quant Plant Biol. 2025 Mar 27;6:e9. doi: 10.1017/qpb.2025.5 (PMC11955309; doi:10.1017/qpb.2025.5)
Supplement: Battu et al. supplementary material [file S2632882825000050sup001.zip › S2632882825000050sup001/BattuFigS2.pdf]

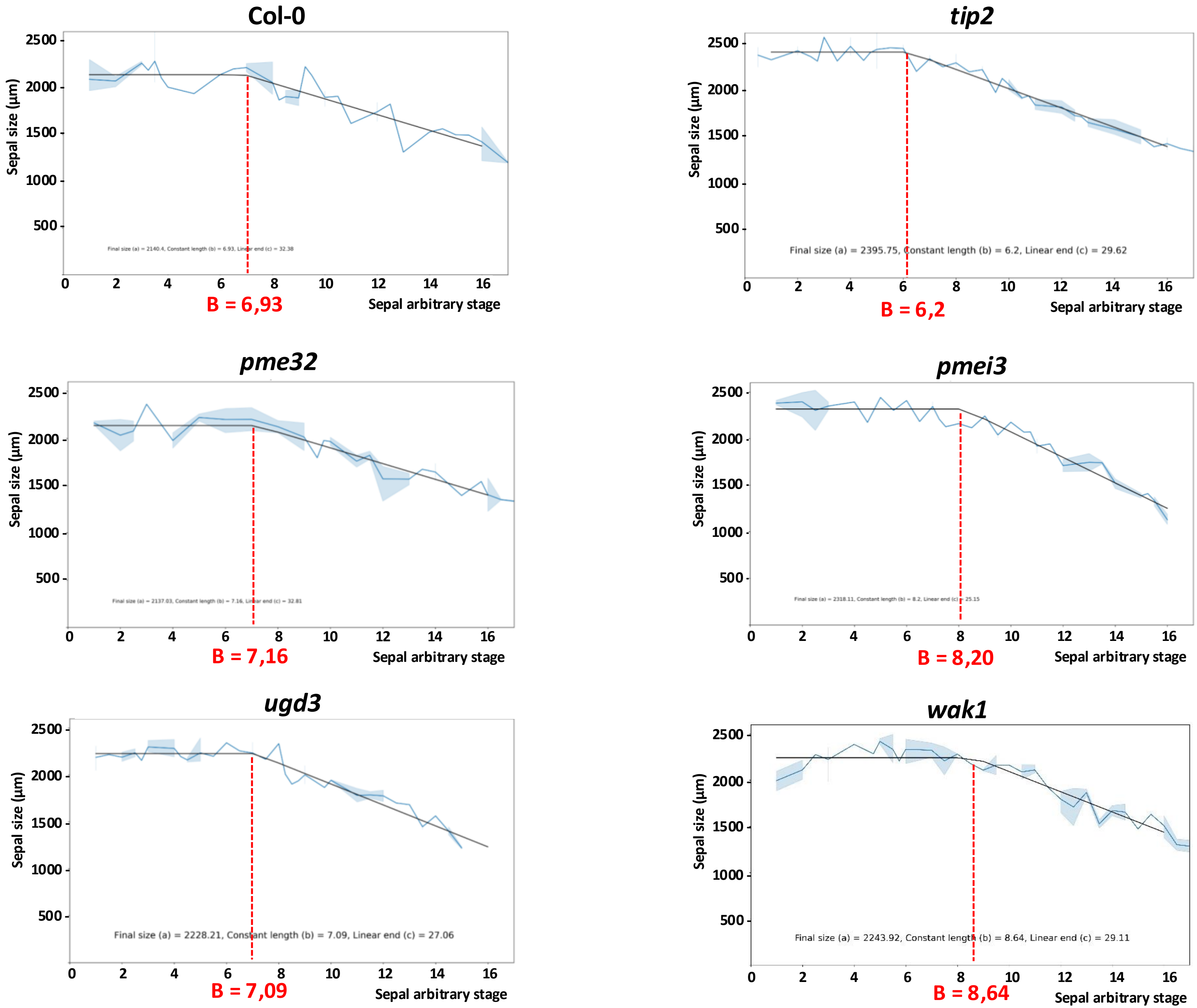

Col-0
Sepal size (µm)
Final size (a) = 2140.4, Constant length (b) = 6.93, Linear end (c) = 32.38
Sepal arbitrary stage
B = 6,93
tip2
Sepal size (µm)
Final size (a) = 2395.75, Constant length (b) = 6.2, Linear end (c) = 29.62
Sepal arbitrary stage
B = 6,2
pme32
Sepal size (µm)
Final size (a) = 2137.03, Constant length (b) = 7.16, Linear end (c) = 32.81
Sepal arbitrary stage
B = 7,16
pmei3
Sepal size (µm)
Final size (a) = 2318.11, Constant length (b) = 8.2, Linear end (c) = 25.15
Sepal arbitrary stage
B = 8,20
ugd3
Sepal size (µm)
Final size (a) = 2228.21, Constant length (b) = 7.09, Linear end (c) = 27.06
Sepal arbitrary stage
B = 7,09
wak1
Sepal size (µm)
Final size (a) = 2243.92, Constant length (b) = 8.64, Linear end (c) = 29.11
Sepal arbitrary stage
B = 8,64

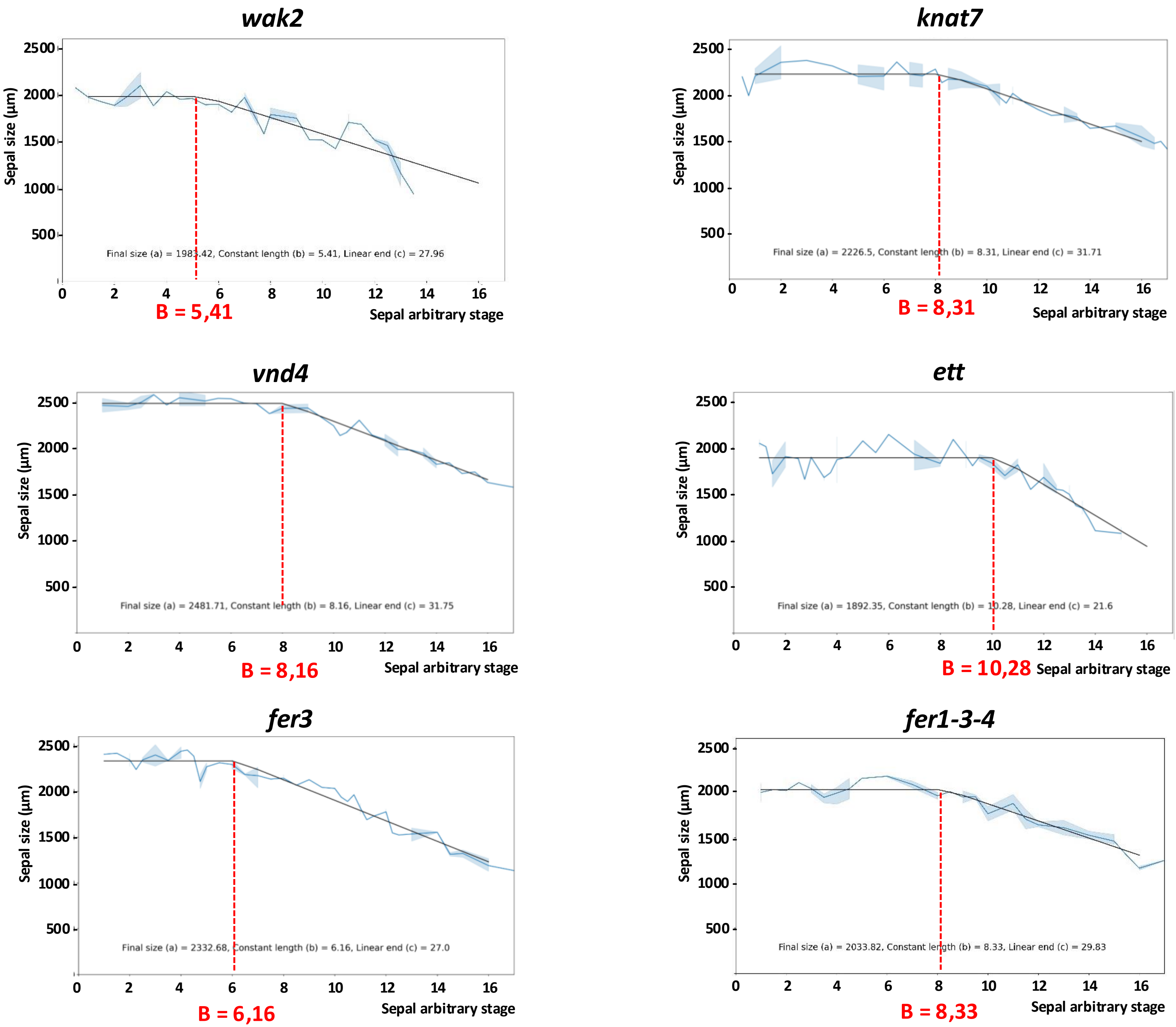
wak2
Sepal size (µm)
Final size (a) = 1983.42, Constant length (b) = 5.41, Linear end (c) = 27.96
B = 5,41
Sepal arbitrary stage
knat7
Sepal size (µm)
Final size (a) = 2226.5, Constant length (b) = 8.31, Linear end (c) = 31.71
B = 8,31
Sepal arbitrary stage
vnd4
Sepal size (µm)
Final size (a) = 2481.71, Constant length (b) = 8.16, Linear end (c) = 31.75
B = 8,16
Sepal arbitrary stage
ett
Sepal size (µm)
Final size (a) = 1892.35, Constant length (b) = 10.28, Linear end (c) = 21.6
B = 10,28
Sepal arbitrary stage
fer3
Sepal size (µm)
Final size (a) = 2332.68, Constant length (b) = 6.16, Linear end (c) = 27.0
B = 6,16
Sepal arbitrary stage
fer1-3-4
Sepal size (µm)
Final size (a) = 2033.82, Constant length (b) = 8.33, Linear end (c) = 29.83
B = 8,33
Sepal arbitrary stage

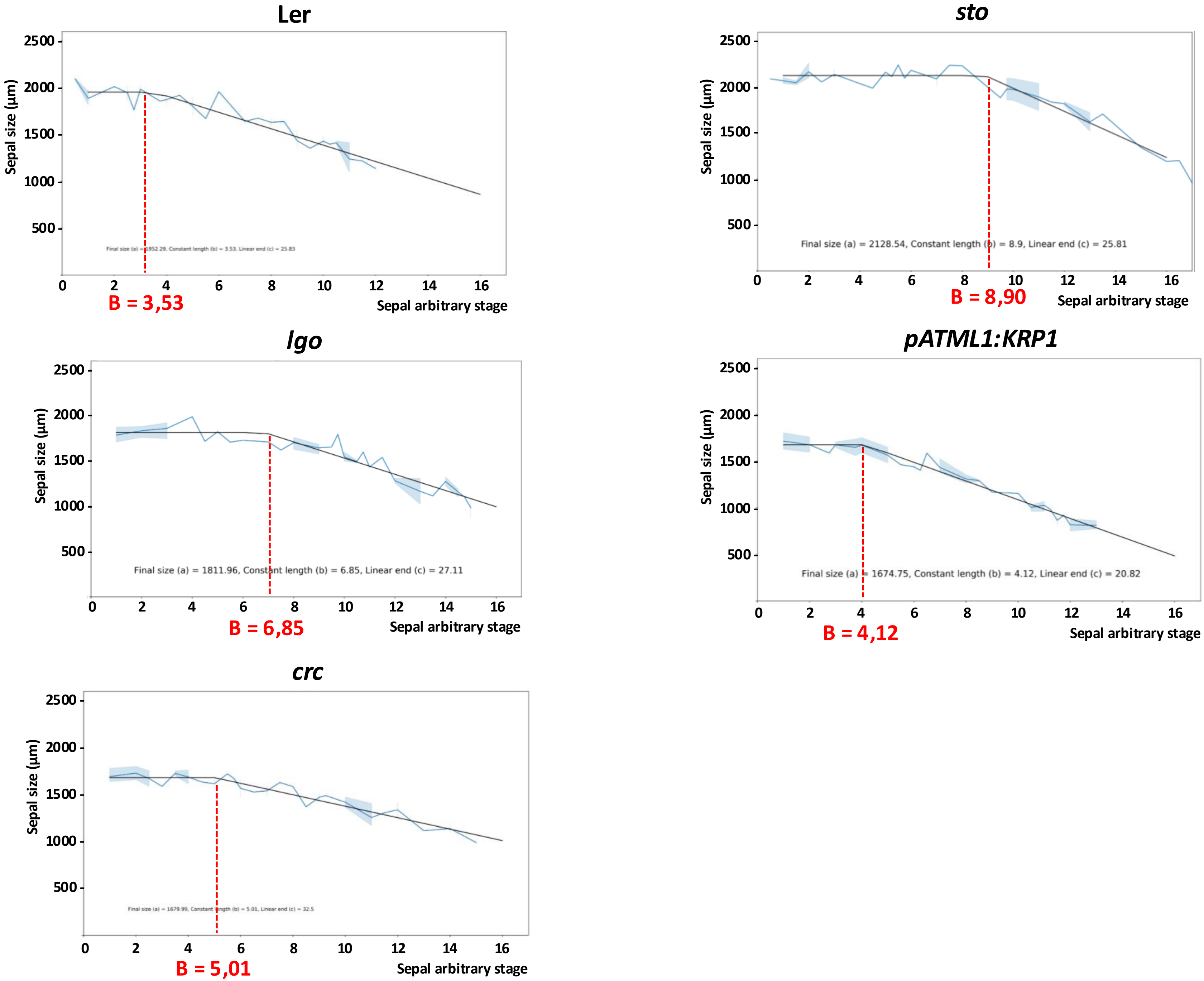
Ler
Sepal size (µm)
Final size (a) = 1952.29, Constant length (b) = 3.53, Linear end (c) = 25.83
B = 3,53
Sepal arbitrary stage
sto
Sepal size (µm)
Final size (a) = 2128.54, Constant length (b) = 8.9, Linear end (c) = 25.81
B = 8,90
Sepal arbitrary stage
lgo
Sepal size (µm)
Final size (a) = 1811.96, Constant length (b) = 6.85, Linear end (c) = 27.11
B = 6,85
Sepal arbitrary stage
pATML1:KRP1
Sepal size (µm)
Final size (a) = 1674.75, Constant length (b) = 4.12, Linear end (c) = 20.82
B = 4,12
Sepal arbitrary stage
crc
Sepal size (µm)
Final size (a) = 1679.99, Constant length (b) = 5.01, Linear end (c) = 32.5
B = 5,01
Sepal arbitrary stage

Figure S2: Sepals growth curves.

For each genotype, we measured abaxial sepal length from all the flowers of an inflorescence, from the oldest fully open flower (arbitrary defined as stage 0) to the youngest flower buds until around 1mm long. We then opened these flowers to visualize internal organs and evaluate the developmental stage. Sepal growth curves were drawn with flowers ordered according to their developmental stage. For each genotype, growth curves were produced by pooling the flowers from four inflorescences. We used the following equation $f(x)=A$ if $x<B$, $f(x)=A(1-(x-B)/(C-B))$ where f represents the sepal curvilinear length, x the flower stage number along the inflorescence (0 being the oldest open flower), A the final curvilinear length of the sepal, B the stage number of the last mature sepal, and C the projected sepal of length 0 following a linear projection. Fits were performed using the "curve_fit" function of the python package "scipy.optimize" (Virtanen 2020). We determined that for all the genotypes analyzed, abaxial sepals from arbitrary stages 1 to 3 (corresponding to open flowers) have reached their final size.
