# Supplementary material for: A 3D morpho-space of sepal geometry reveals the importance of organ curvature
Source: Quant Plant Biol. 2025 Mar 27;6:e9. doi: 10.1017/qpb.2025.5 (PMC11955309; doi:10.1017/qpb.2025.5)
Supplement: Battu et al. supplementary material [file S2632882825000050sup001.zip › S2632882825000050sup001/BattuFigS3.pdf]

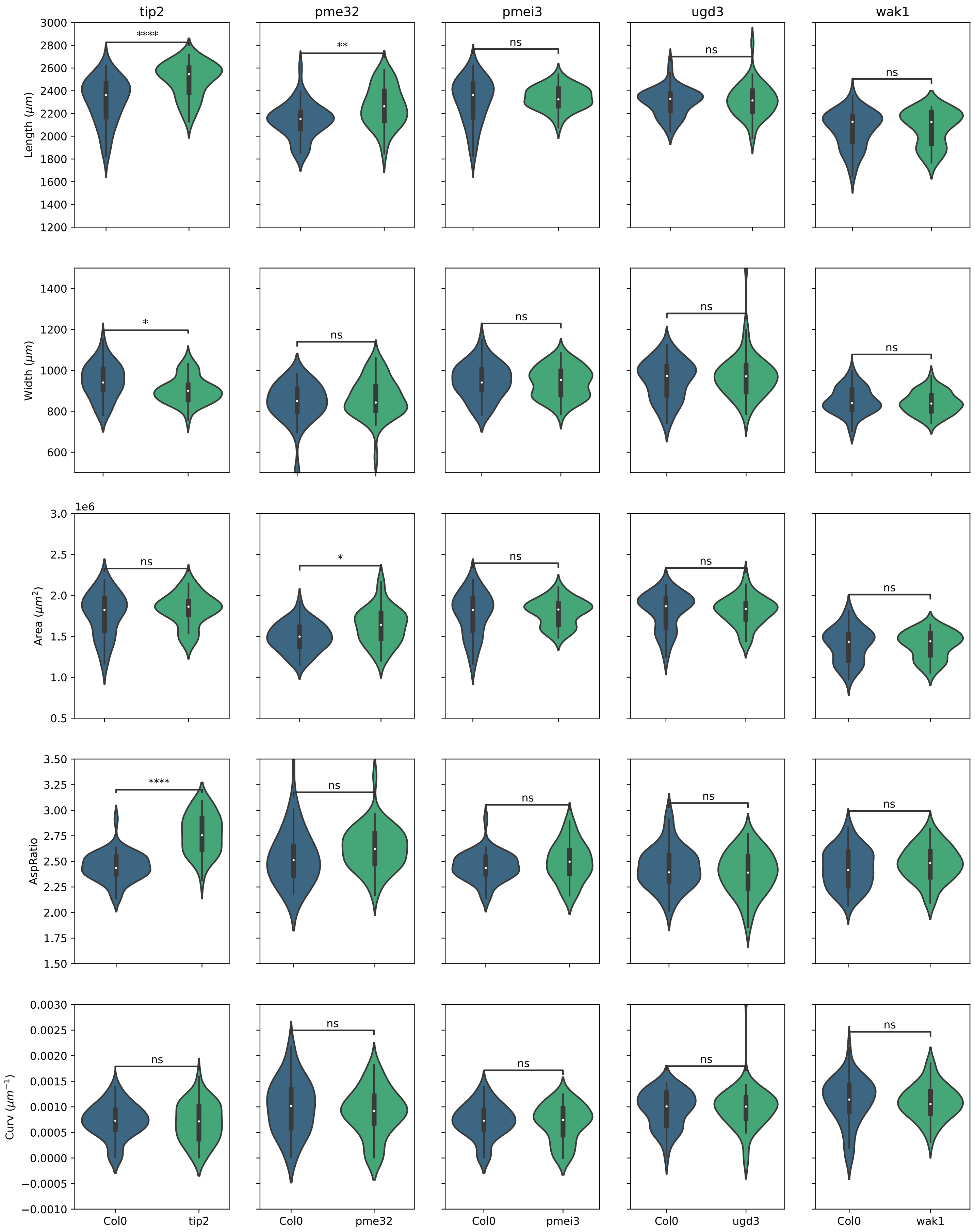

tip2
pme32
pmei3
ugd3
wak1
Length (μm)
Width (μm)
Area (μm^2)
AspRatio
Curv (μm^{-1})
1e6
****
**
ns
*
Col0
tip2
pme32
pmei3
ugd3
wak1

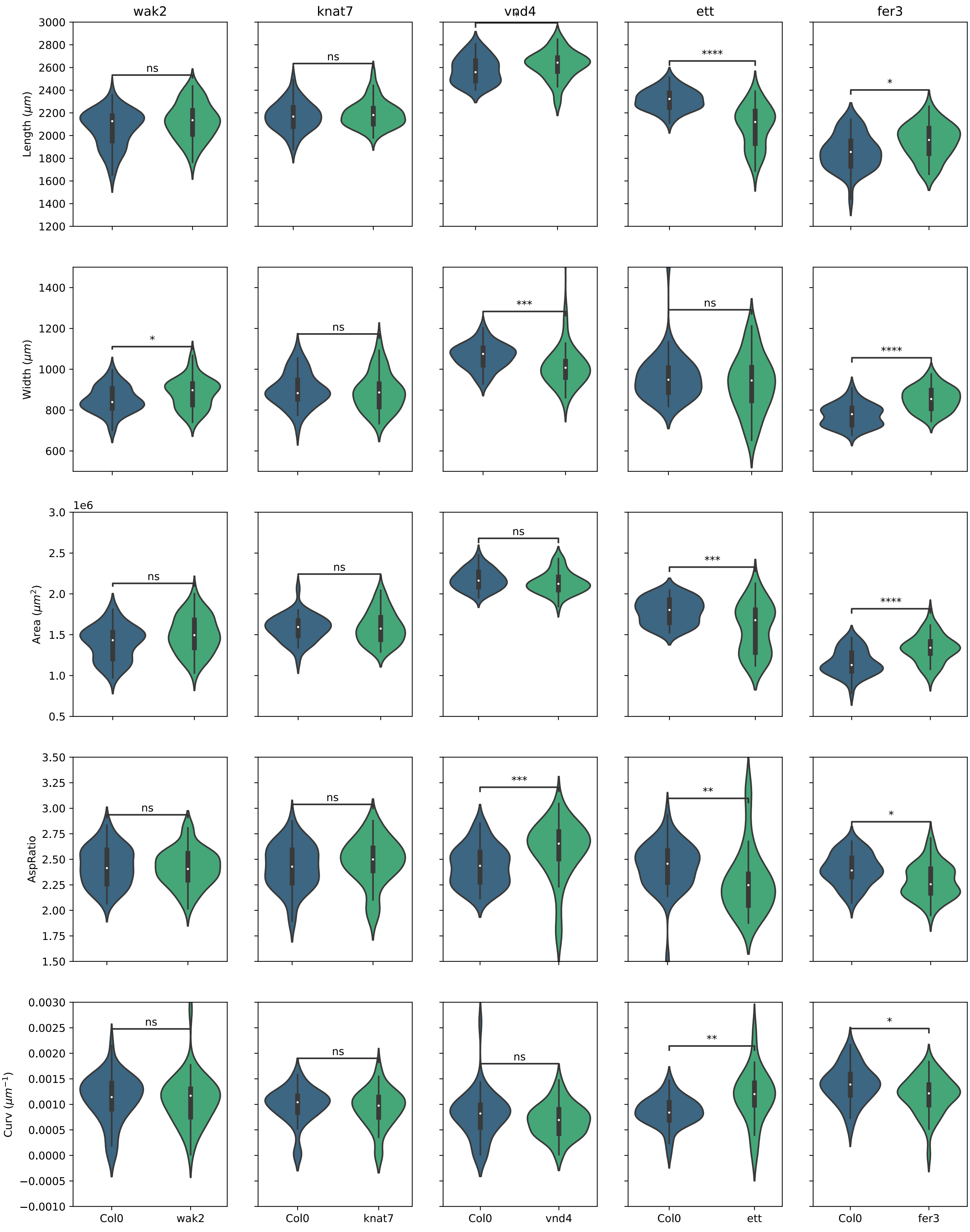
wak2
knat7
vnd4
ett
fer3
Length (μm)
Width (μm)
Area (μm^2)
1e6
AspRatio
Curv (μm^{-1})
ns
*
**
***
****
Col0
wak2
knat7
vnd4
ett
fer3

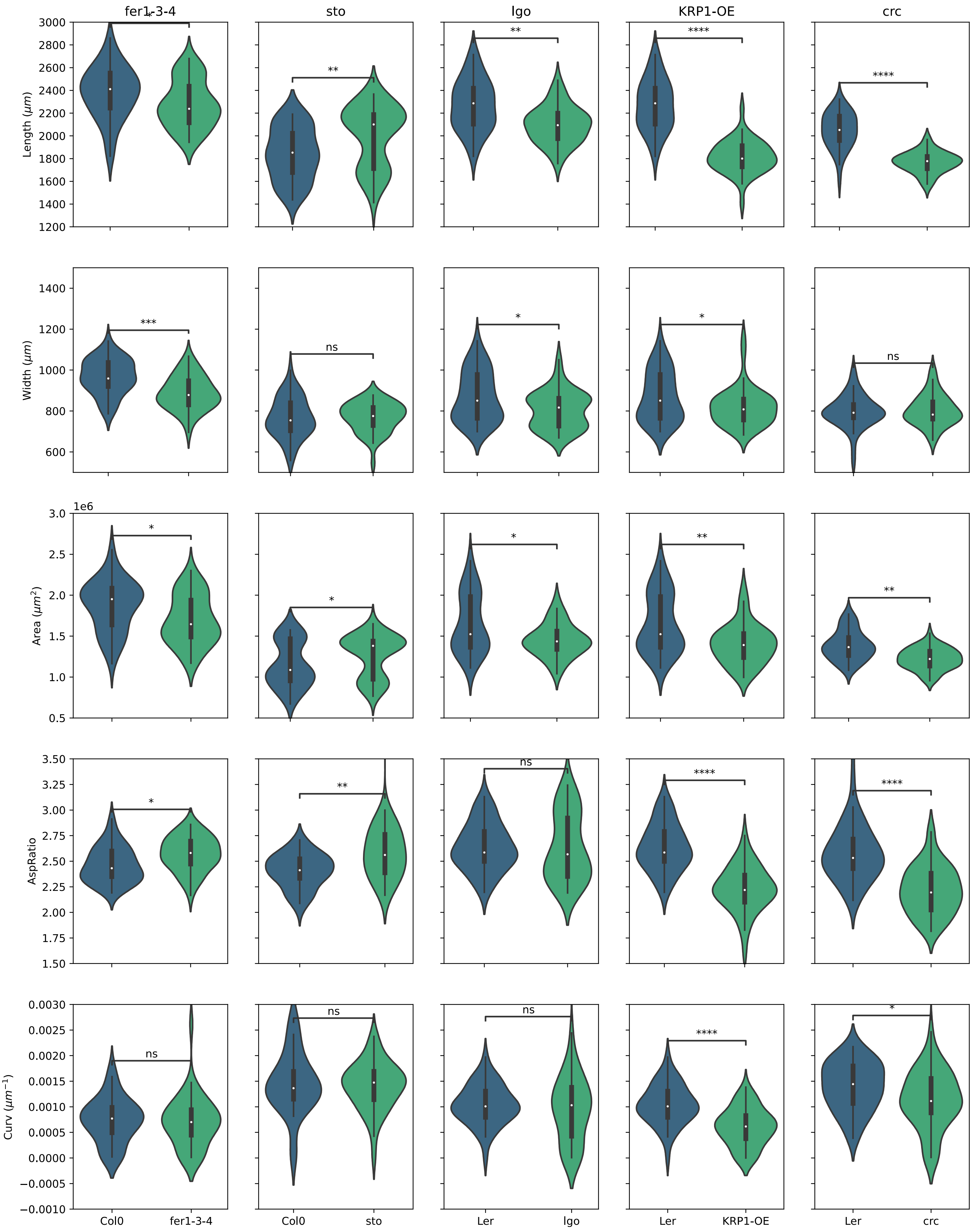

fer1-3-4
sto
lgo
KRP1-OE
crc
Length (μm)
Width (μm)
Area (μm²)
1e6
AspRatio
Curv (μm⁻¹)
*
**
***
****
ns
Col0
fer1-3-4
Col0
sto
Ler
lgo
Ler
KRP1-OE
Ler
crc

Figure S3. Violin plots. Measured morphological data (Length, Width, Area, Aspect ratio and Curvature) are represented on violin plots for each mutant and its corresponding wild-type. The result for Kolmogorov-Smirnov test for comparing the two distributions is noted on each plot: ns indicates that the difference between the distributions of the mutant and wild-type samples is not significant (p-value>0.05), while significance is denoted by stars in the following way: * if 0.005<p-value<=0.05, ** if 0.0005<p-value<=0.005, *** if 0.00005<p-value<=0.0005 and **** if p-value<=0.00005. Test results are summarized in table 2 of the main text.
