# Supplementary material for: A 3D morpho-space of sepal geometry reveals the importance of organ curvature
Source: Quant Plant Biol. 2025 Mar 27;6:e9. doi: 10.1017/qpb.2025.5 (PMC11955309; doi:10.1017/qpb.2025.5)
Supplement: Battu et al. supplementary material [file S2632882825000050sup001.zip › S2632882825000050sup001/BattuFigS4.pdf]

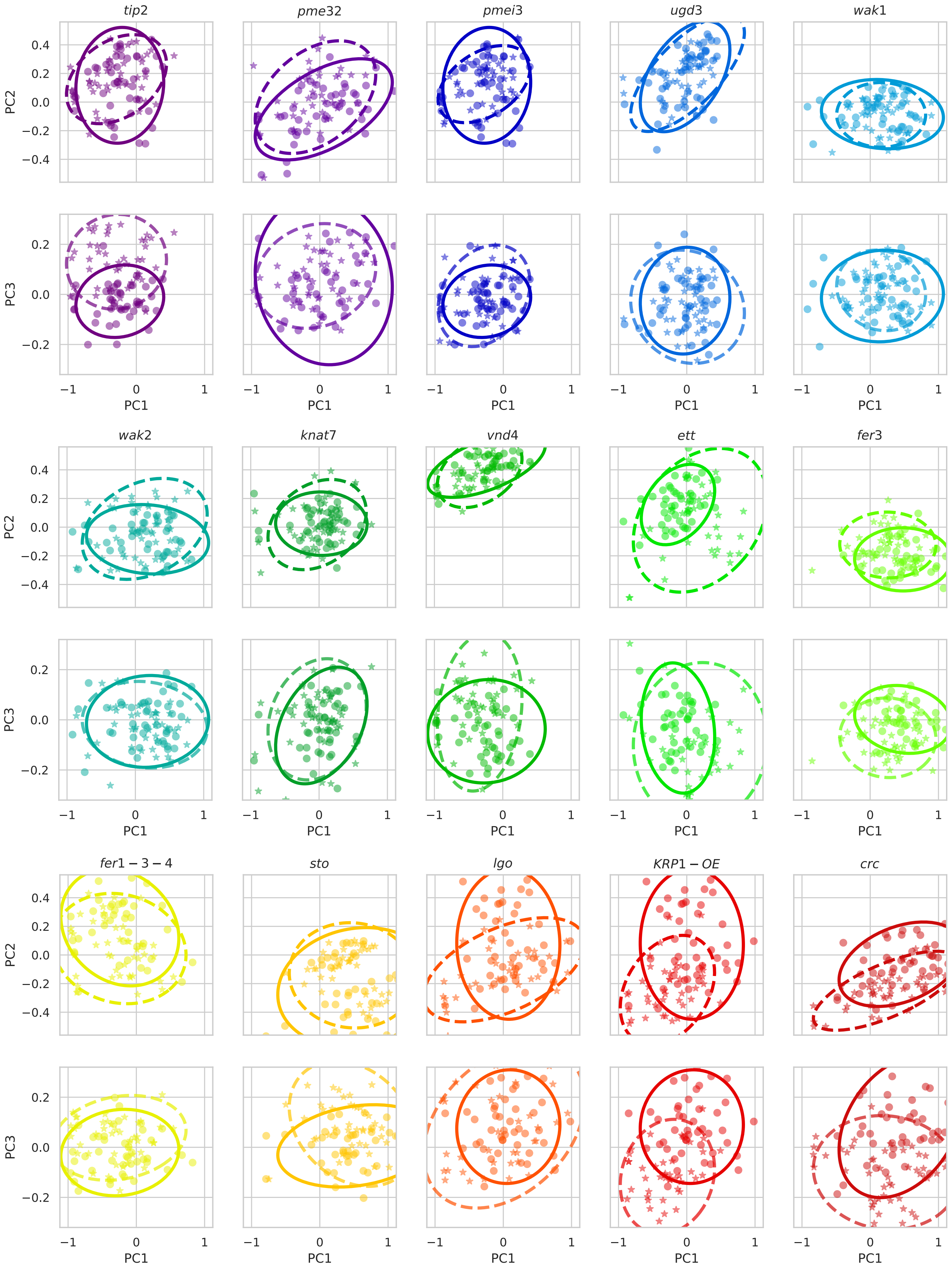

tip2
pme32
pmei3
ugd3
wak1
wak2
knat7
vnd4
ett
fer3
fer1 − 3 − 4
sto
lgo
KRP1 − OE
crc
PC1
PC2
PC3
0.4
0.2
0.0
−0.2
−0.4
−1
0
1

Figure S4. Pairwise comparison of the mutants with their wild-type controls. All mutant/wild-type pairs (different colours) are represented in the common PCA space. Continuous ellipses correspond to confidence region (semi-axes corresponding to 20% of the standard deviation) of wild-type sepals marked by dots, while dashed ellipses correspond to confidence regions of mutant sepals marked by stars. Confident ellipses have semi-axis of twice the standard deviation.
