# Supplementary material for: A 3D morpho-space of sepal geometry reveals the importance of organ curvature
Source: Quant Plant Biol. 2025 Mar 27;6:e9. doi: 10.1017/qpb.2025.5 (PMC11955309; doi:10.1017/qpb.2025.5)
Supplement: Battu et al. supplementary material [file S2632882825000050sup001.zip › S2632882825000050sup001/BattuFigS5.pdf]

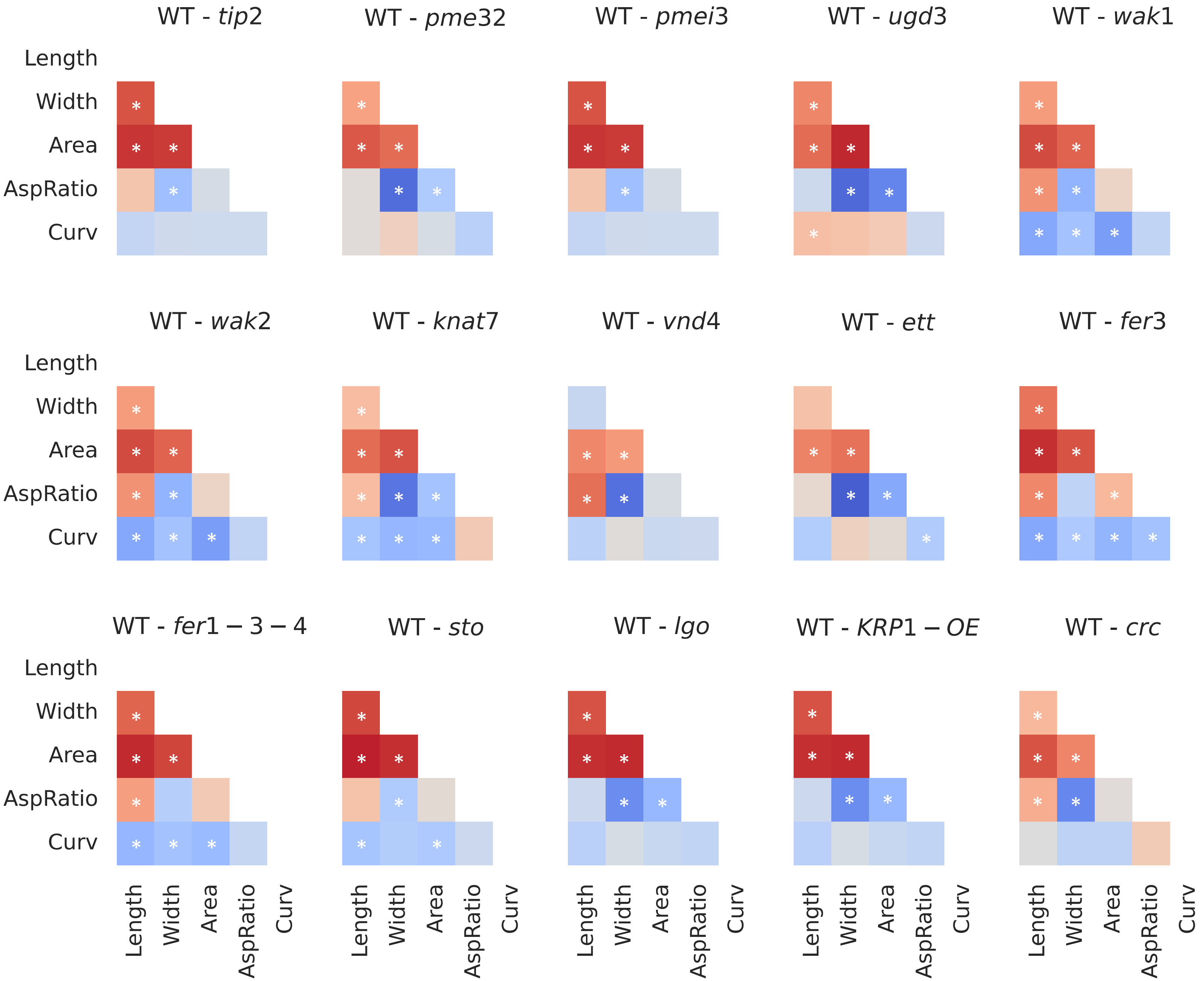

Figure S5. Correlation heat-map of morphological parameters for wild-type samples. The common colour bar represents Pearson correlation coefficients, an asterisk is placed on significant correlation values (having a p-value>0.05 when testing for non-correlation).
