# Supplementary material for: A 3D morpho-space of sepal geometry reveals the importance of organ curvature
Source: Quant Plant Biol. 2025 Mar 27;6:e9. doi: 10.1017/qpb.2025.5 (PMC11955309; doi:10.1017/qpb.2025.5)
Supplement: Battu et al. supplementary material [file S2632882825000050sup001.zip › S2632882825000050sup001/BattuFigS6.pdf]

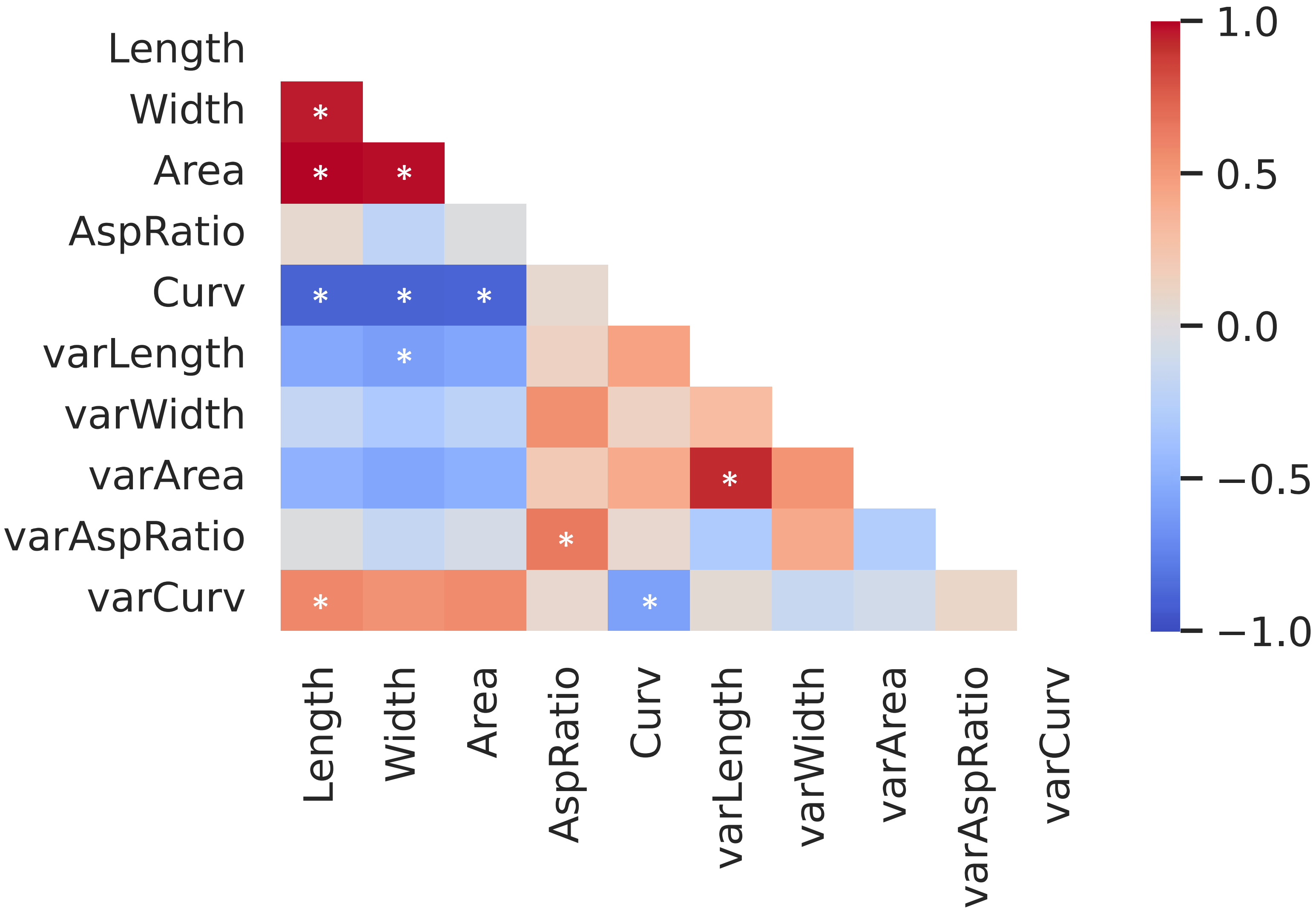

Figure S6. Inter experiment correlation heat-map of morphological parameters and their variabilities for wild-type samples. We consider the collection of all available wild-type samples (N=12), where each individual is characterised by the means as well as the variability of the measured parameters. Colour bar represents Pearson correlation coefficients, an asterisk is placed on significant correlation values (having a p-value>0.05 when testing for non-correlation).
