# Supplementary material for: A 3D morpho-space of sepal geometry reveals the importance of organ curvature
Source: Quant Plant Biol. 2025 Mar 27;6:e9. doi: 10.1017/qpb.2025.5 (PMC11955309; doi:10.1017/qpb.2025.5)
Supplement: Battu et al. supplementary material [file S2632882825000050sup001.zip › S2632882825000050sup001/FigureS1.pptx]

## Slide 1
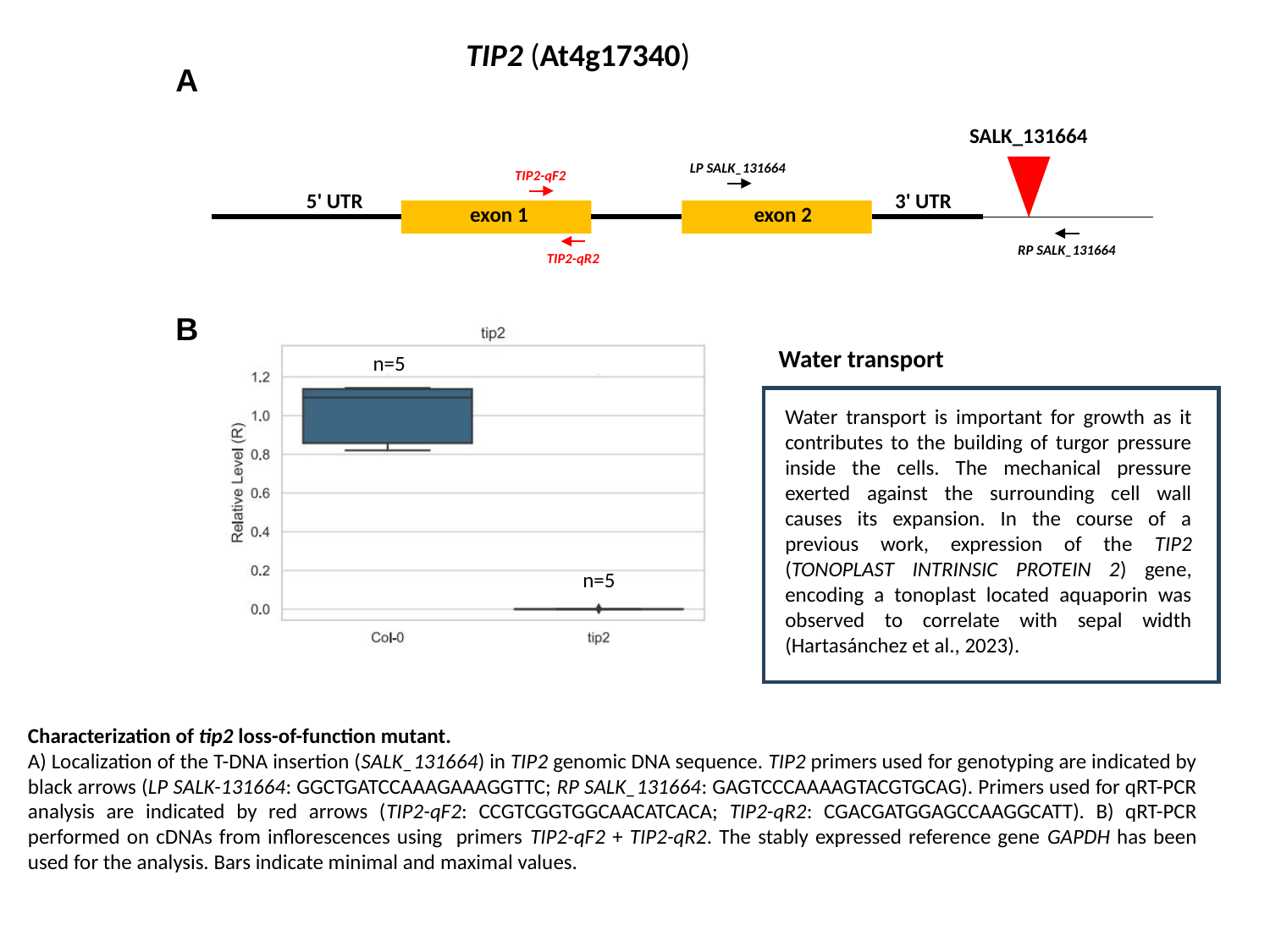

TIP2 (At4g17340)
A
SALK_131664
LP SALK_131664
TIP2-qF2
5' UTR
3' UTR
exon 2
exon 1
RP SALK_131664
TIP2-qR2
B
Characterization of tip2 loss-of-function mutant.
A) Localization of the T-DNA insertion (SALK_131664) in TIP2 genomic DNA sequence. TIP2 primers used for genotyping are indicated by black arrows (LP SALK-131664: GGCTGATCCAAAGAAAGGTTC; RP SALK_131664: GAGTCCCAAAAGTACGTGCAG). Primers used for qRT-PCR analysis are indicated by red arrows (TIP2-qF2: CCGTCGGTGGCAACATCACA; TIP2-qR2: CGACGATGGAGCCAAGGCATT). B) qRT-PCR performed on cDNAs from inflorescences using primers TIP2-qF2 + TIP2-qR2. The stably expressed reference gene GAPDH has been used for the analysis. Bars indicate minimal and maximal values.
n=5
n=5
Water transport
Water transport is important for growth as it contributes to the building of turgor pressure inside the cells. The mechanical pressure exerted against the surrounding cell wall causes its expansion. In the course of a previous work, expression of the TIP2 (TONOPLAST INTRINSIC PROTEIN 2) gene, encoding a tonoplast located aquaporin was observed to correlate with sepal width (Hartasánchez et al., 2023).

## Slide 2
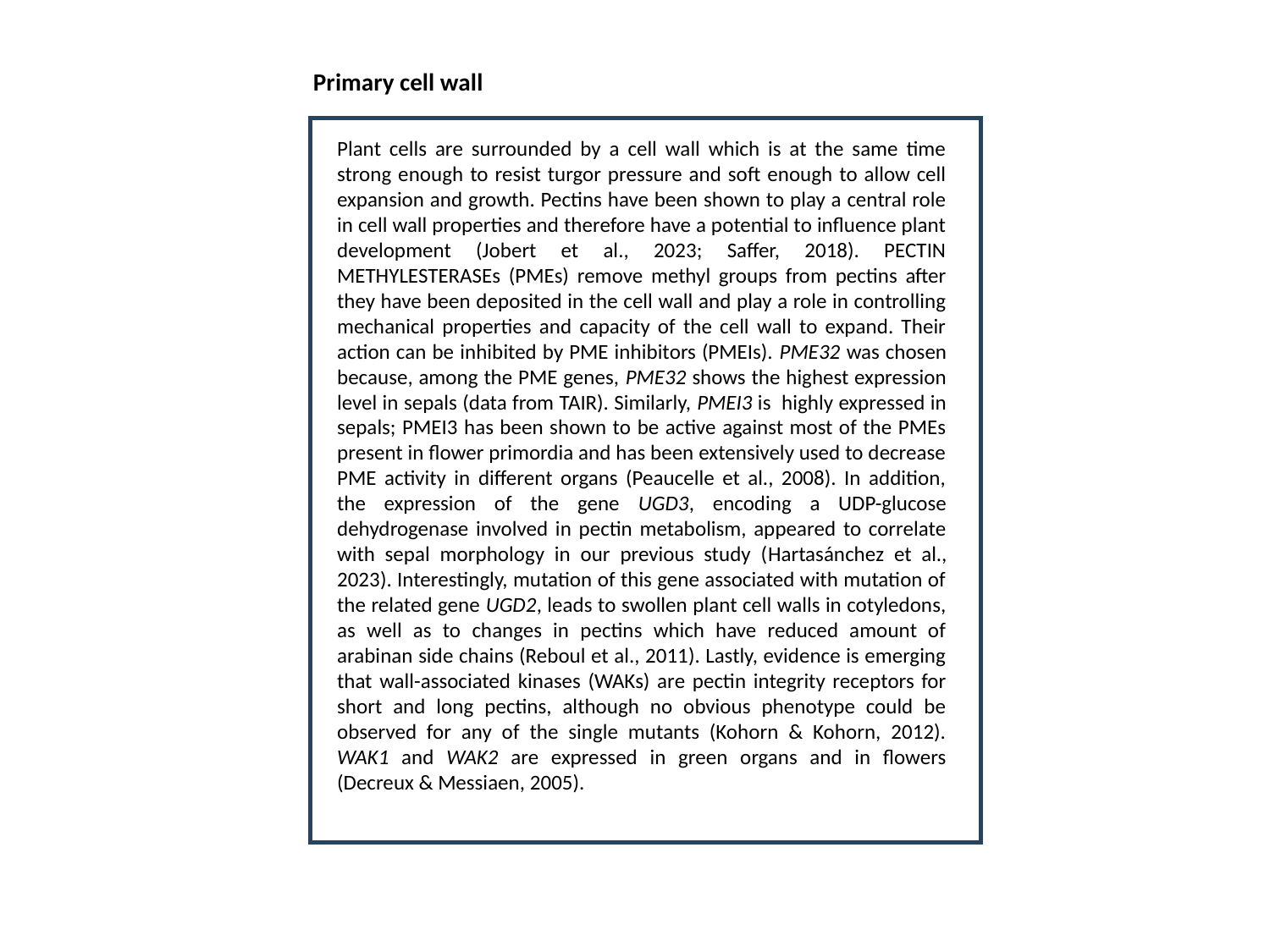

Primary cell wall
Plant cells are surrounded by a cell wall which is at the same time strong enough to resist turgor pressure and soft enough to allow cell expansion and growth. Pectins have been shown to play a central role in cell wall properties and therefore have a potential to influence plant development (Jobert et al., 2023; Saffer, 2018). PECTIN METHYLESTERASEs (PMEs) remove methyl groups from pectins after they have been deposited in the cell wall and play a role in controlling mechanical properties and capacity of the cell wall to expand. Their action can be inhibited by PME inhibitors (PMEIs). PME32 was chosen because, among the PME genes, PME32 shows the highest expression level in sepals (data from TAIR). Similarly, PMEI3 is highly expressed in sepals; PMEI3 has been shown to be active against most of the PMEs present in flower primordia and has been extensively used to decrease PME activity in different organs (Peaucelle et al., 2008). In addition, the expression of the gene UGD3, encoding a UDP-glucose dehydrogenase involved in pectin metabolism, appeared to correlate with sepal morphology in our previous study (Hartasánchez et al., 2023). Interestingly, mutation of this gene associated with mutation of the related gene UGD2, leads to swollen plant cell walls in cotyledons, as well as to changes in pectins which have reduced amount of arabinan side chains (Reboul et al., 2011). Lastly, evidence is emerging that wall-associated kinases (WAKs) are pectin integrity receptors for short and long pectins, although no obvious phenotype could be observed for any of the single mutants (Kohorn & Kohorn, 2012). WAK1 and WAK2 are expressed in green organs and in flowers (Decreux & Messiaen, 2005).

## Slide 3
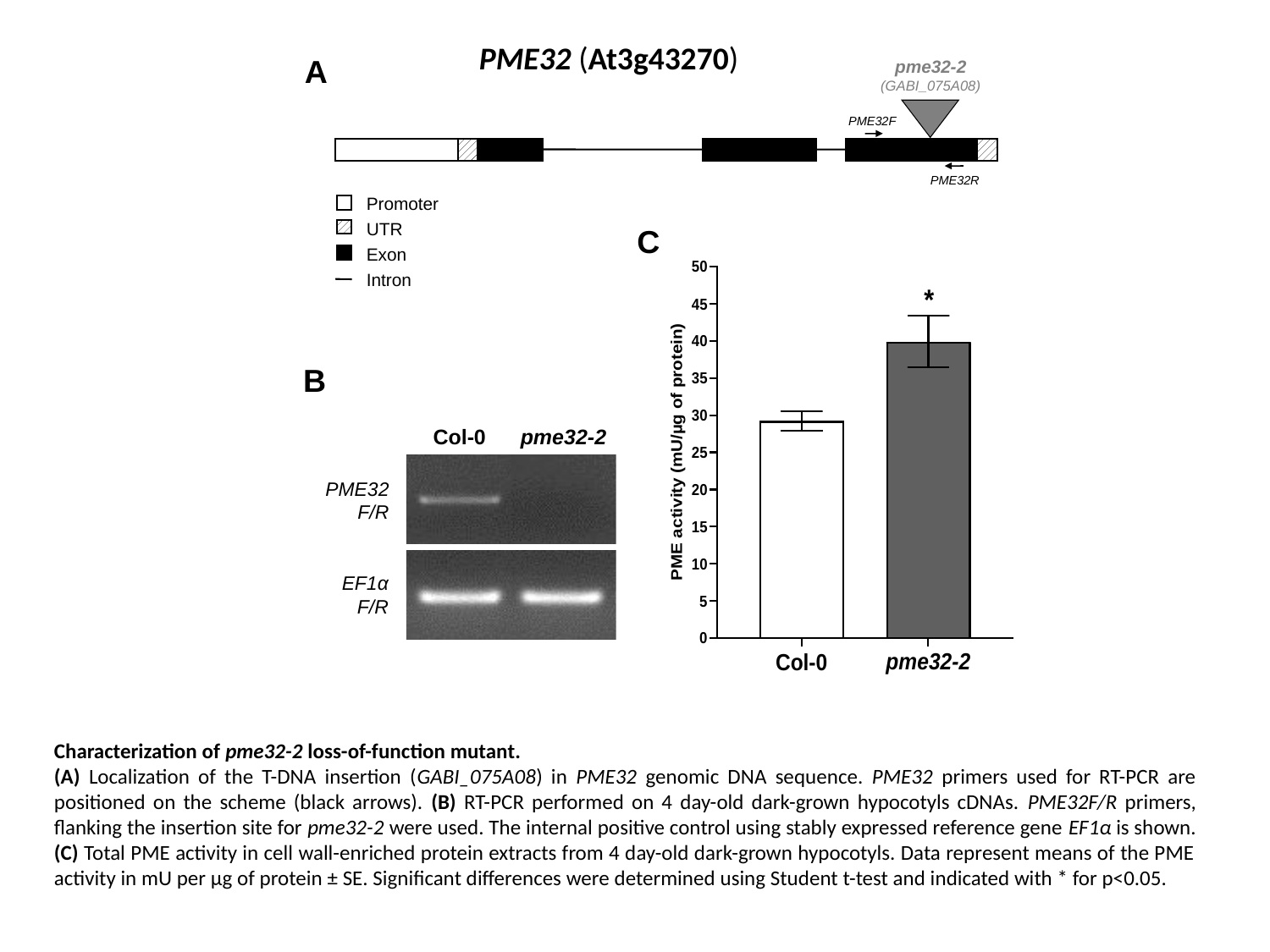

PME32 (At3g43270)
A
pme32-2
(GABI_075A08)
PME32F
PME32R
Promoter
UTR
Exon
Intron
C
B
Col-0
pme32-2
PME32
F/R
EF1α
F/R
Characterization of pme32-2 loss-of-function mutant.
(A) Localization of the T-DNA insertion (GABI_075A08) in PME32 genomic DNA sequence. PME32 primers used for RT-PCR are positioned on the scheme (black arrows). (B) RT-PCR performed on 4 day-old dark-grown hypocotyls cDNAs. PME32F/R primers, flanking the insertion site for pme32-2 were used. The internal positive control using stably expressed reference gene EF1α is shown. (C) Total PME activity in cell wall-enriched protein extracts from 4 day-old dark-grown hypocotyls. Data represent means of the PME activity in mU per µg of protein ± SE. Significant differences were determined using Student t-test and indicated with * for p<0.05.

## Slide 4
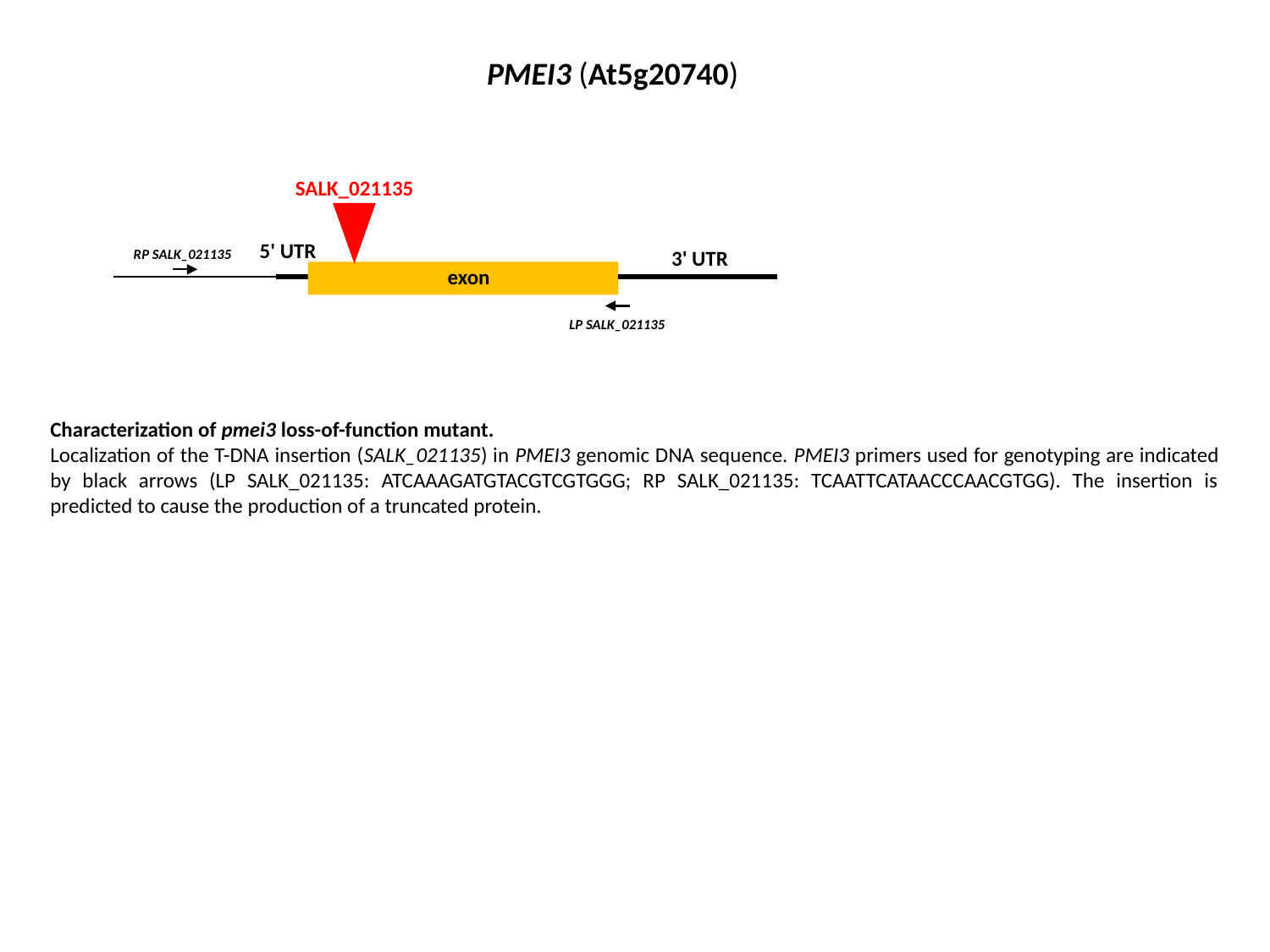

PMEI3 (At5g20740)
SALK_021135
5' UTR
RP SALK_021135
3' UTR
exon
LP SALK_021135
Characterization of pmei3 loss-of-function mutant.
Localization of the T-DNA insertion (SALK_021135) in PMEI3 genomic DNA sequence. PMEI3 primers used for genotyping are indicated by black arrows (LP SALK_021135: ATCAAAGATGTACGTCGTGGG; RP SALK_021135: TCAATTCATAACCCAACGTGG). The insertion is predicted to cause the production of a truncated protein.

## Slide 5
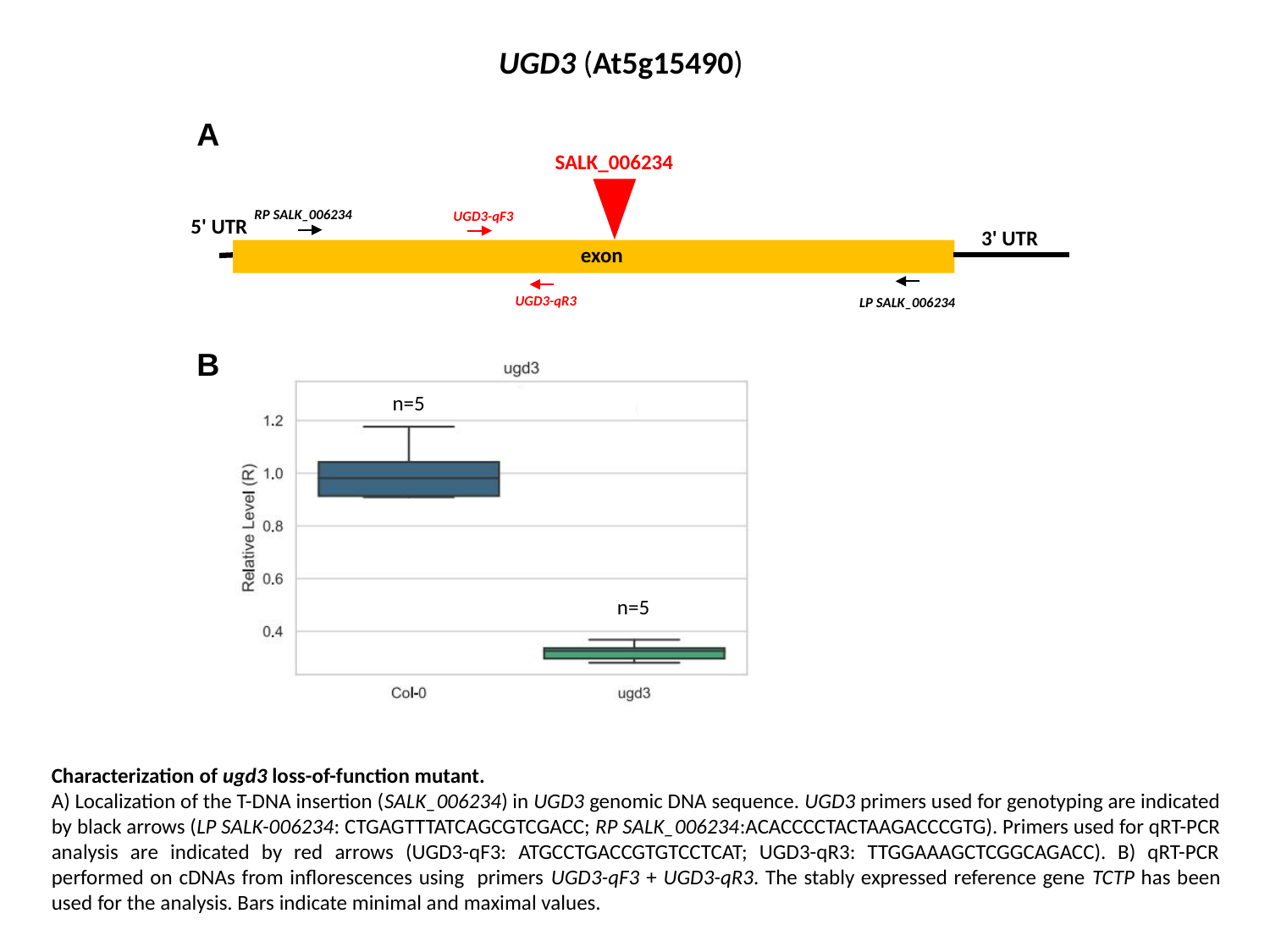

UGD3 (At5g15490)
A
SALK_006234
RP SALK_006234
UGD3-qF3
5' UTR
3' UTR
exon
LP SALK_006234
UGD3-qR3
B
Characterization of ugd3 loss-of-function mutant.
A) Localization of the T-DNA insertion (SALK_006234) in UGD3 genomic DNA sequence. UGD3 primers used for genotyping are indicated by black arrows (LP SALK-006234: CTGAGTTTATCAGCGTCGACC; RP SALK_006234:ACACCCCTACTAAGACCCGTG). Primers used for qRT-PCR analysis are indicated by red arrows (UGD3-qF3: ATGCCTGACCGTGTCCTCAT; UGD3-qR3: TTGGAAAGCTCGGCAGACC). B) qRT-PCR performed on cDNAs from inflorescences using primers UGD3-qF3 + UGD3-qR3. The stably expressed reference gene TCTP has been used for the analysis. Bars indicate minimal and maximal values.
n=5
n=5

## Slide 6
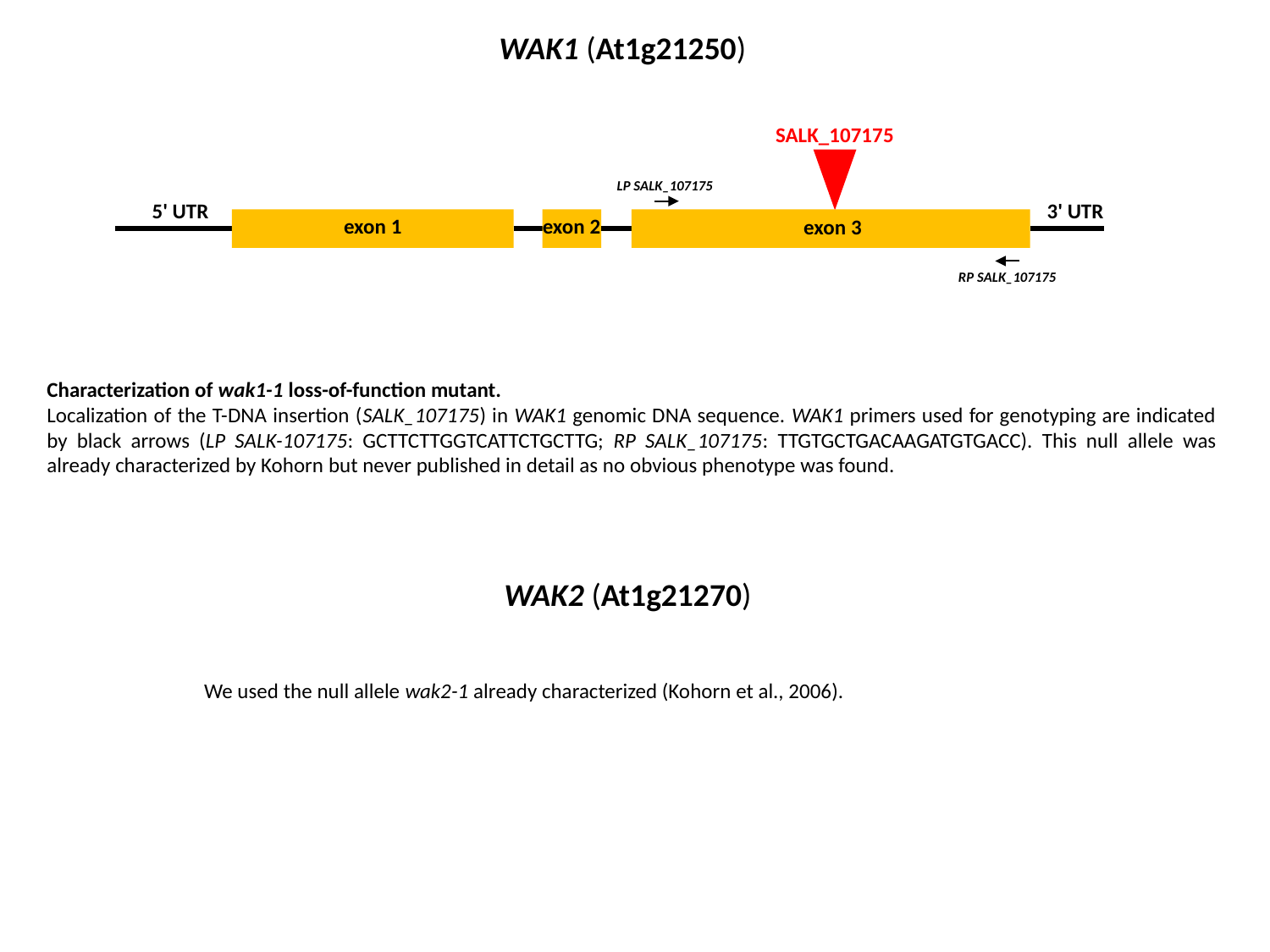

WAK1 (At1g21250)
SALK_107175
LP SALK_107175
5' UTR
3' UTR
exon 2
exon 1
exon 3
RP SALK_107175
Characterization of wak1-1 loss-of-function mutant.
Localization of the T-DNA insertion (SALK_107175) in WAK1 genomic DNA sequence. WAK1 primers used for genotyping are indicated by black arrows (LP SALK-107175: GCTTCTTGGTCATTCTGCTTG; RP SALK_107175: TTGTGCTGACAAGATGTGACC). This null allele was already characterized by Kohorn but never published in detail as no obvious phenotype was found.
WAK2 (At1g21270)
We used the null allele wak2-1 already characterized (Kohorn et al., 2006).

## Slide 7
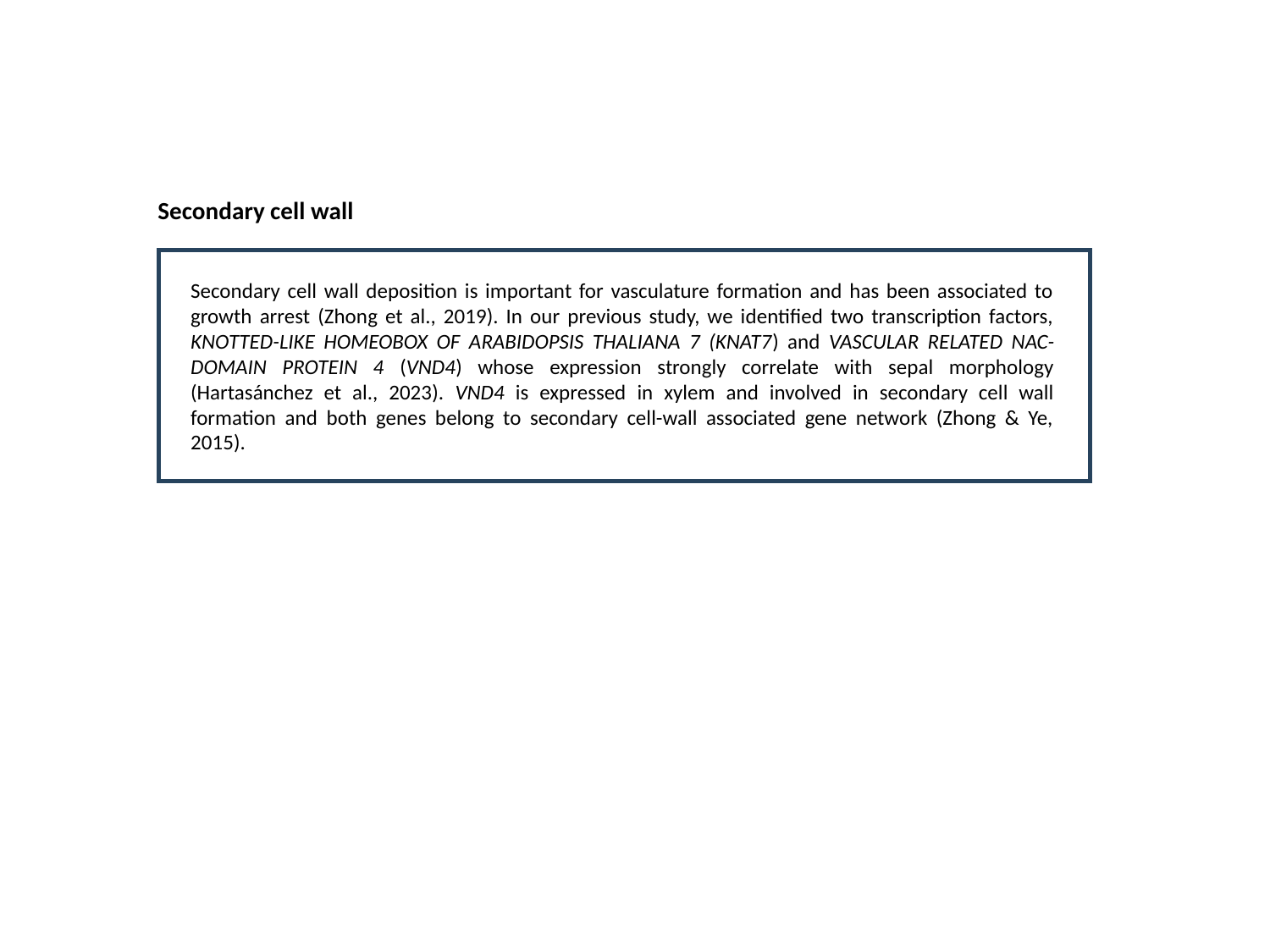

Secondary cell wall
Secondary cell wall deposition is important for vasculature formation and has been associated to growth arrest (Zhong et al., 2019). In our previous study, we identified two transcription factors, KNOTTED-LIKE HOMEOBOX OF ARABIDOPSIS THALIANA 7 (KNAT7) and VASCULAR RELATED NAC-DOMAIN PROTEIN 4 (VND4) whose expression strongly correlate with sepal morphology (Hartasánchez et al., 2023). VND4 is expressed in xylem and involved in secondary cell wall formation and both genes belong to secondary cell-wall associated gene network (Zhong & Ye, 2015).

## Slide 8
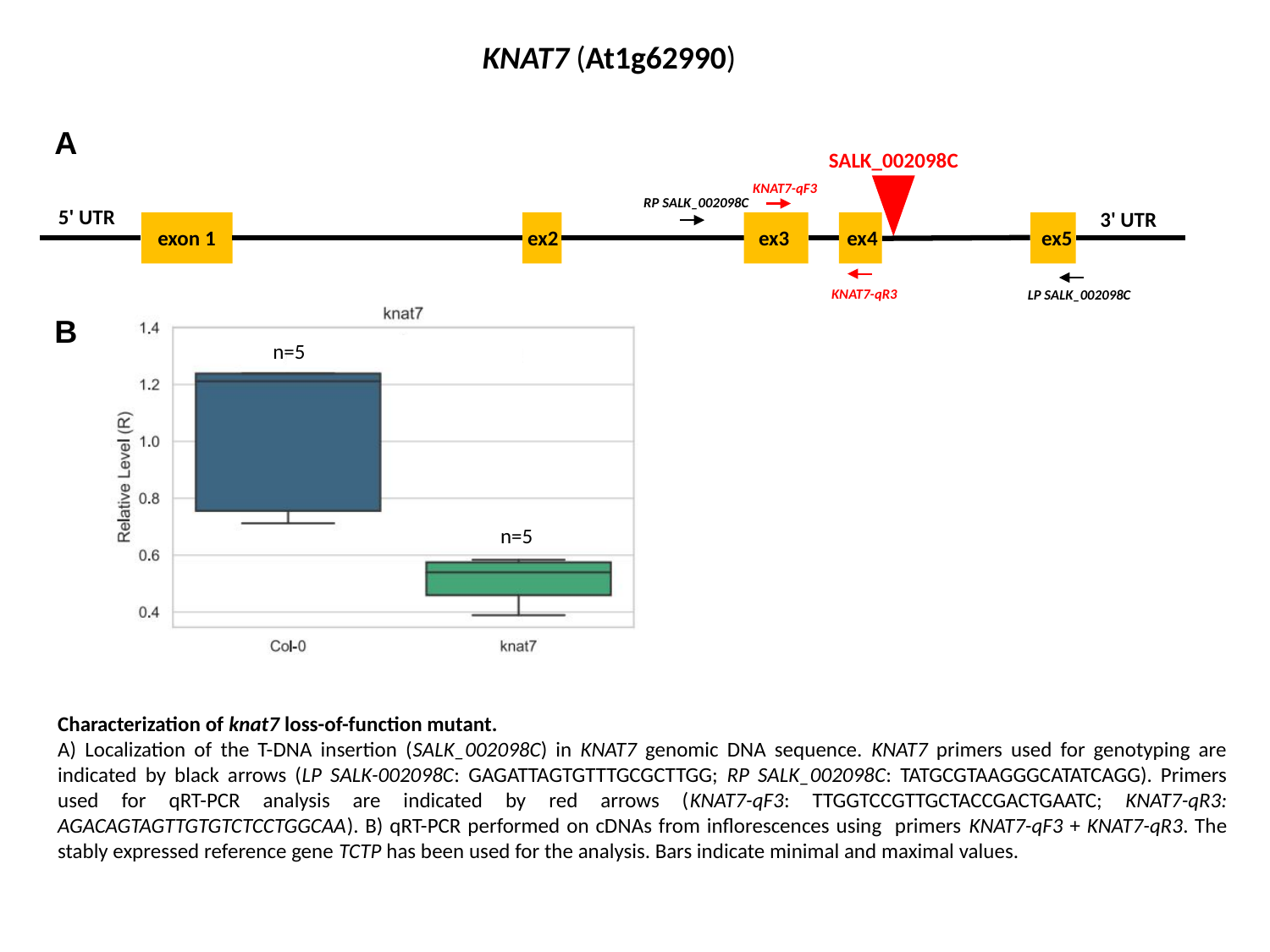

KNAT7 (At1g62990)
A
SALK_002098C
KNAT7-qF3
RP SALK_002098C
5' UTR
3' UTR
exon 1
ex2
ex3
ex4
ex5
KNAT7-qR3
LP SALK_002098C
B
Characterization of knat7 loss-of-function mutant.
A) Localization of the T-DNA insertion (SALK_002098C) in KNAT7 genomic DNA sequence. KNAT7 primers used for genotyping are indicated by black arrows (LP SALK-002098C: GAGATTAGTGTTTGCGCTTGG; RP SALK_002098C: TATGCGTAAGGGCATATCAGG). Primers used for qRT-PCR analysis are indicated by red arrows (KNAT7-qF3: TTGGTCCGTTGCTACCGACTGAATC; KNAT7-qR3: AGACAGTAGTTGTGTCTCCTGGCAA). B) qRT-PCR performed on cDNAs from inflorescences using primers KNAT7-qF3 + KNAT7-qR3. The stably expressed reference gene TCTP has been used for the analysis. Bars indicate minimal and maximal values.
n=5
n=5

## Slide 9
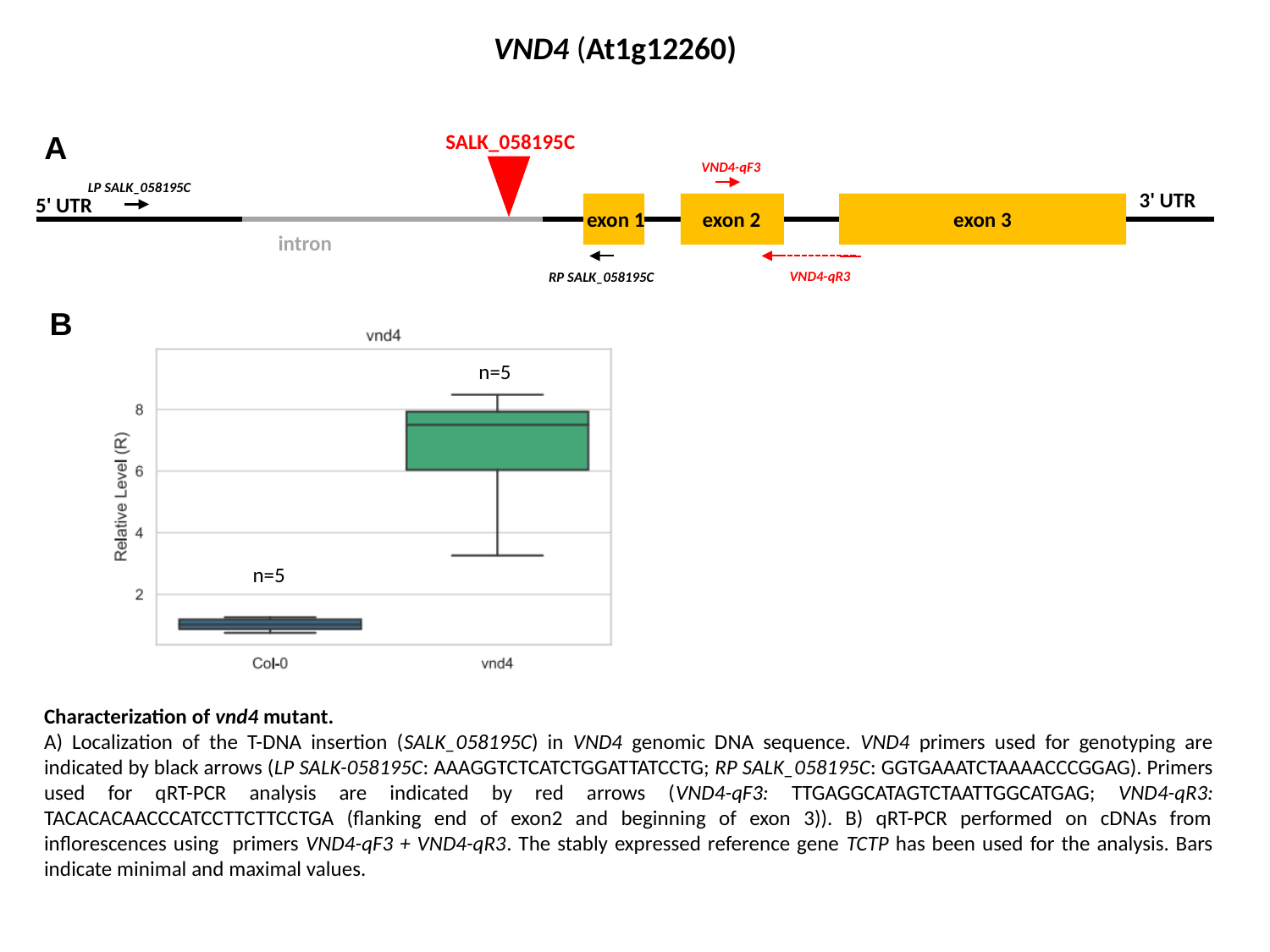

VND4 (At1g12260)
SALK_058195C
LP SALK_058195C
3' UTR
5' UTR
exon 1
exon 2
exon 3
intron
RP SALK_058195C
A
VND4-qF3
VND4-qR3
B
Characterization of vnd4 mutant.
A) Localization of the T-DNA insertion (SALK_058195C) in VND4 genomic DNA sequence. VND4 primers used for genotyping are indicated by black arrows (LP SALK-058195C: AAAGGTCTCATCTGGATTATCCTG; RP SALK_058195C: GGTGAAATCTAAAACCCGGAG). Primers used for qRT-PCR analysis are indicated by red arrows (VND4-qF3: TTGAGGCATAGTCTAATTGGCATGAG; VND4-qR3: TACACACAACCCATCCTTCTTCCTGA (flanking end of exon2 and beginning of exon 3)). B) qRT-PCR performed on cDNAs from inflorescences using primers VND4-qF3 + VND4-qR3. The stably expressed reference gene TCTP has been used for the analysis. Bars indicate minimal and maximal values.
n=5
n=5

## Slide 10
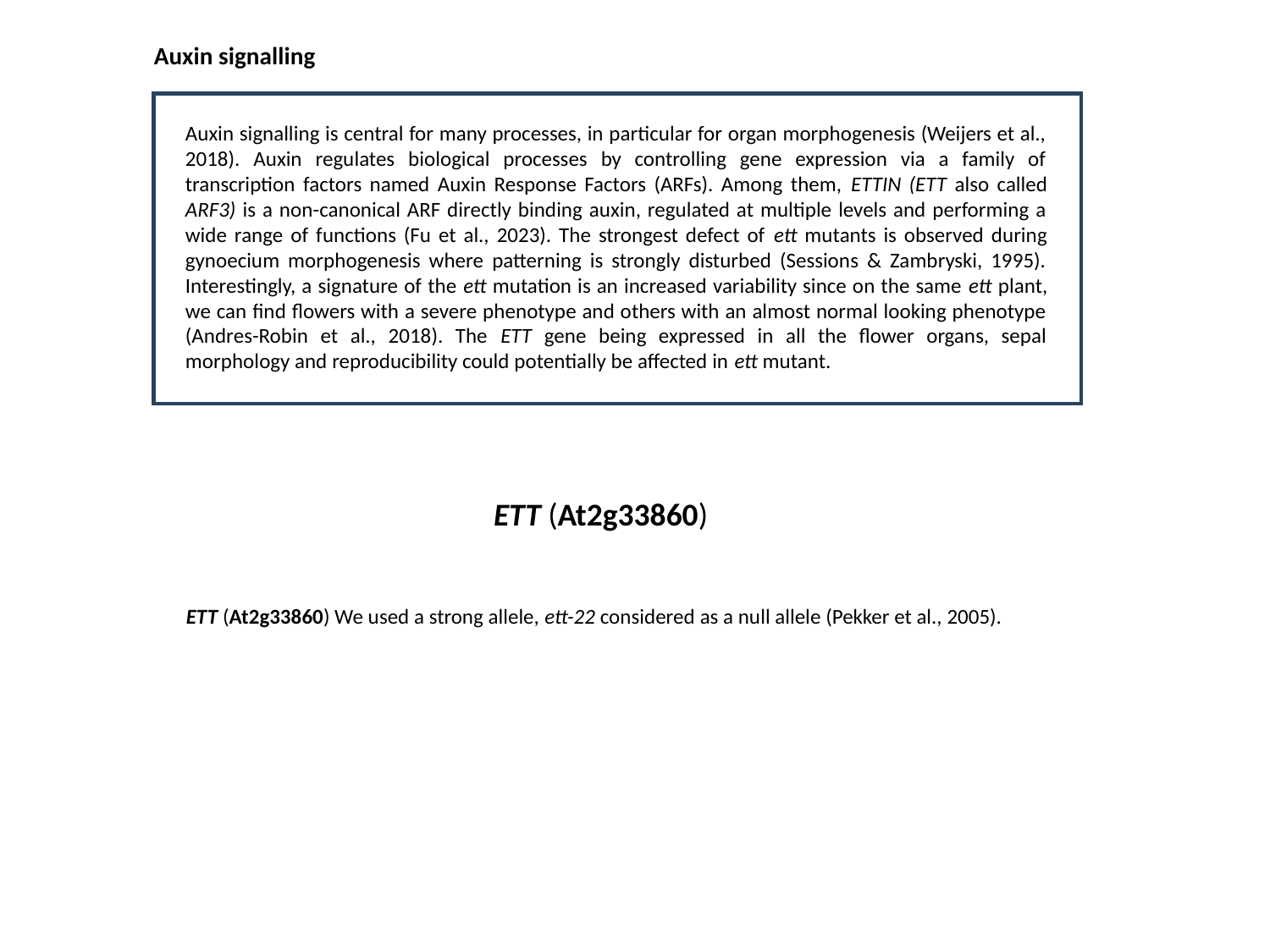

Auxin signalling
Auxin signalling is central for many processes, in particular for organ morphogenesis (Weijers et al., 2018). Auxin regulates biological processes by controlling gene expression via a family of transcription factors named Auxin Response Factors (ARFs). Among them, ETTIN (ETT also called ARF3) is a non-canonical ARF directly binding auxin, regulated at multiple levels and performing a wide range of functions (Fu et al., 2023). The strongest defect of ett mutants is observed during gynoecium morphogenesis where patterning is strongly disturbed (Sessions & Zambryski, 1995). Interestingly, a signature of the ett mutation is an increased variability since on the same ett plant, we can find flowers with a severe phenotype and others with an almost normal looking phenotype (Andres-Robin et al., 2018). The ETT gene being expressed in all the flower organs, sepal morphology and reproducibility could potentially be affected in ett mutant.
ETT (At2g33860)
ETT (At2g33860) We used a strong allele, ett-22 considered as a null allele (Pekker et al., 2005).

## Slide 11
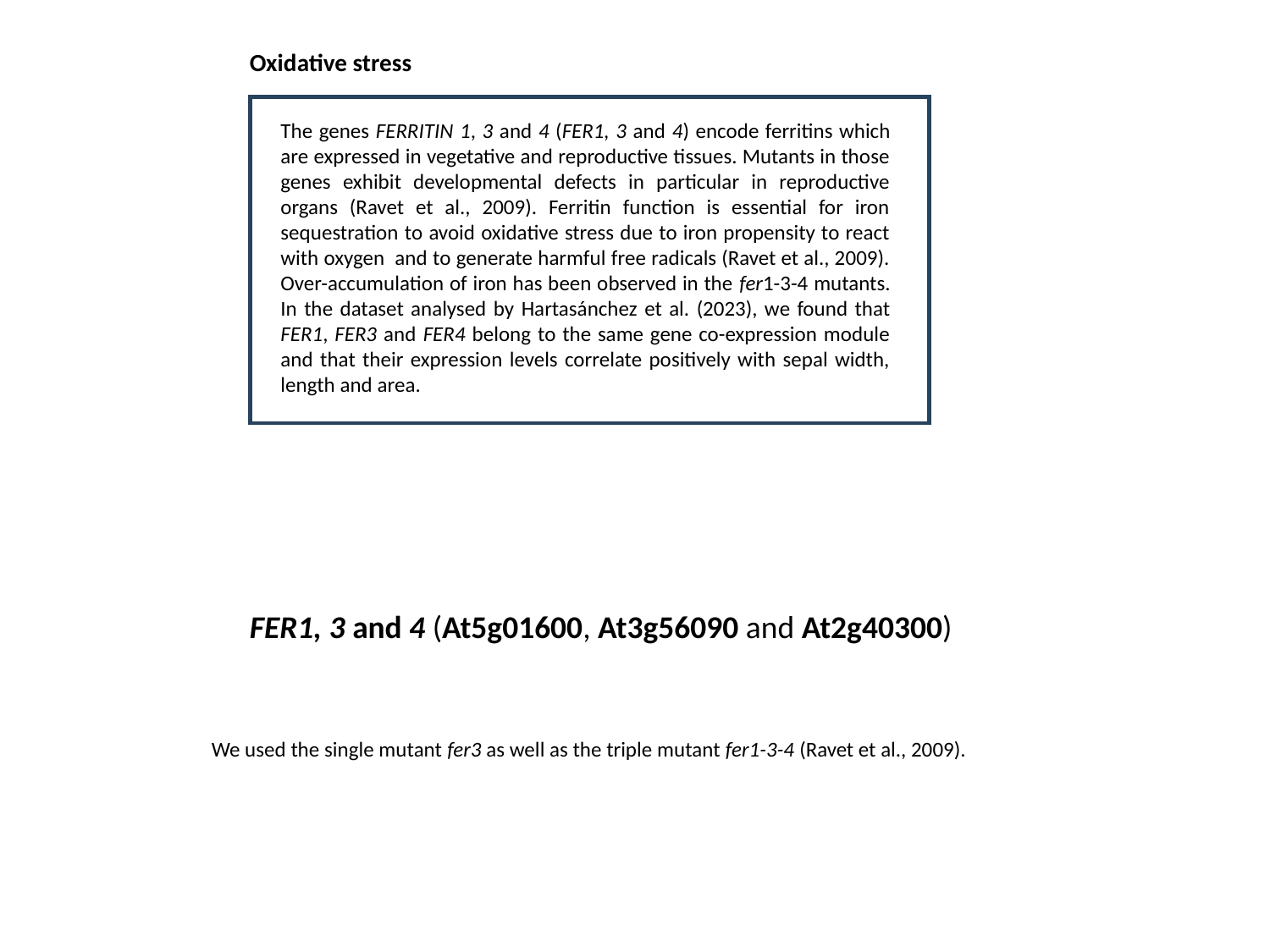

Oxidative stress
The genes FERRITIN 1, 3 and 4 (FER1, 3 and 4) encode ferritins which are expressed in vegetative and reproductive tissues. Mutants in those genes exhibit developmental defects in particular in reproductive organs (Ravet et al., 2009). Ferritin function is essential for iron sequestration to avoid oxidative stress due to iron propensity to react with oxygen  and to generate harmful free radicals (Ravet et al., 2009). Over-accumulation of iron has been observed in the fer1-3-4 mutants. In the dataset analysed by Hartasánchez et al. (2023), we found that FER1, FER3 and FER4 belong to the same gene co-expression module and that their expression levels correlate positively with sepal width, length and area.
FER1, 3 and 4 (At5g01600, At3g56090 and At2g40300)
We used the single mutant fer3 as well as the triple mutant fer1-3-4 (Ravet et al., 2009).

## Slide 12
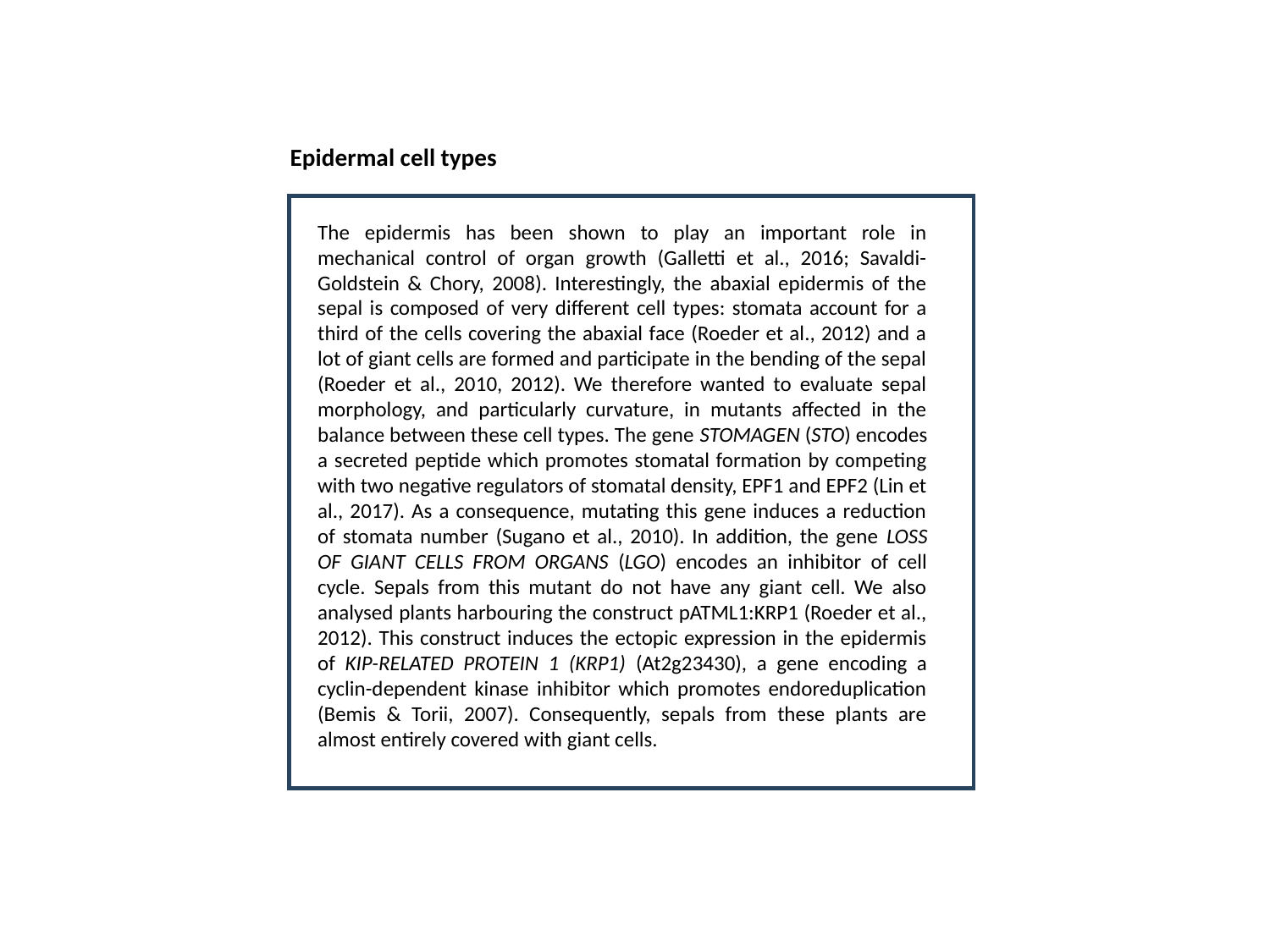

Epidermal cell types
The epidermis has been shown to play an important role in mechanical control of organ growth (Galletti et al., 2016; Savaldi-Goldstein & Chory, 2008). Interestingly, the abaxial epidermis of the sepal is composed of very different cell types: stomata account for a third of the cells covering the abaxial face (Roeder et al., 2012) and a lot of giant cells are formed and participate in the bending of the sepal (Roeder et al., 2010, 2012). We therefore wanted to evaluate sepal morphology, and particularly curvature, in mutants affected in the balance between these cell types. The gene STOMAGEN (STO) encodes a secreted peptide which promotes stomatal formation by competing with two negative regulators of stomatal density, EPF1 and EPF2 (Lin et al., 2017). As a consequence, mutating this gene induces a reduction of stomata number (Sugano et al., 2010). In addition, the gene LOSS OF GIANT CELLS FROM ORGANS (LGO) encodes an inhibitor of cell cycle. Sepals from this mutant do not have any giant cell. We also analysed plants harbouring the construct pATML1:KRP1 (Roeder et al., 2012). This construct induces the ectopic expression in the epidermis of KIP-RELATED PROTEIN 1 (KRP1) (At2g23430), a gene encoding a cyclin-dependent kinase inhibitor which promotes endoreduplication (Bemis & Torii, 2007). Consequently, sepals from these plants are almost entirely covered with giant cells.

## Slide 13
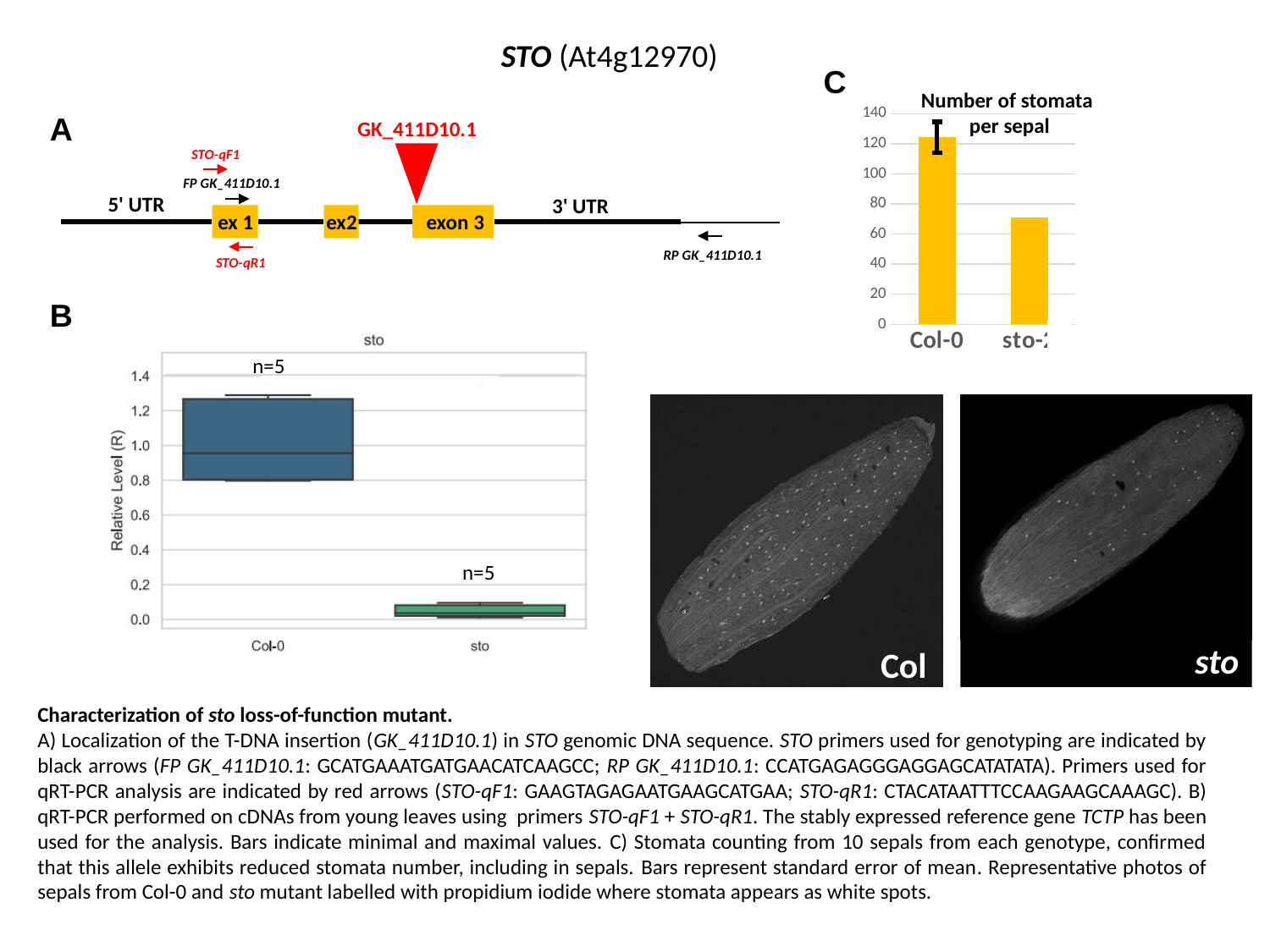

STO (At4g12970)
C
Number of stomata
per sepal
### Chart
| Category | |
|---|---|
| Col-0 | 124.2 |
| sto-2 | 71.0 |A
GK_411D10.1
5' UTR
3' UTR
ex2
ex 1
exon 3
STO-qF1
FP GK_411D10.1
RP GK_411D10.1
STO-qR1
B
sto
Col-0
Characterization of sto loss-of-function mutant.
A) Localization of the T-DNA insertion (GK_411D10.1) in STO genomic DNA sequence. STO primers used for genotyping are indicated by black arrows (FP GK_411D10.1: GCATGAAATGATGAACATCAAGCC; RP GK_411D10.1: CCATGAGAGGGAGGAGCATATATA). Primers used for qRT-PCR analysis are indicated by red arrows (STO-qF1: GAAGTAGAGAATGAAGCATGAA; STO-qR1: CTACATAATTTCCAAGAAGCAAAGC). B) qRT-PCR performed on cDNAs from young leaves using primers STO-qF1 + STO-qR1. The stably expressed reference gene TCTP has been used for the analysis. Bars indicate minimal and maximal values. C) Stomata counting from 10 sepals from each genotype, confirmed that this allele exhibits reduced stomata number, including in sepals. Bars represent standard error of mean. Representative photos of sepals from Col-0 and sto mutant labelled with propidium iodide where stomata appears as white spots.
n=5
n=5

## Slide 14
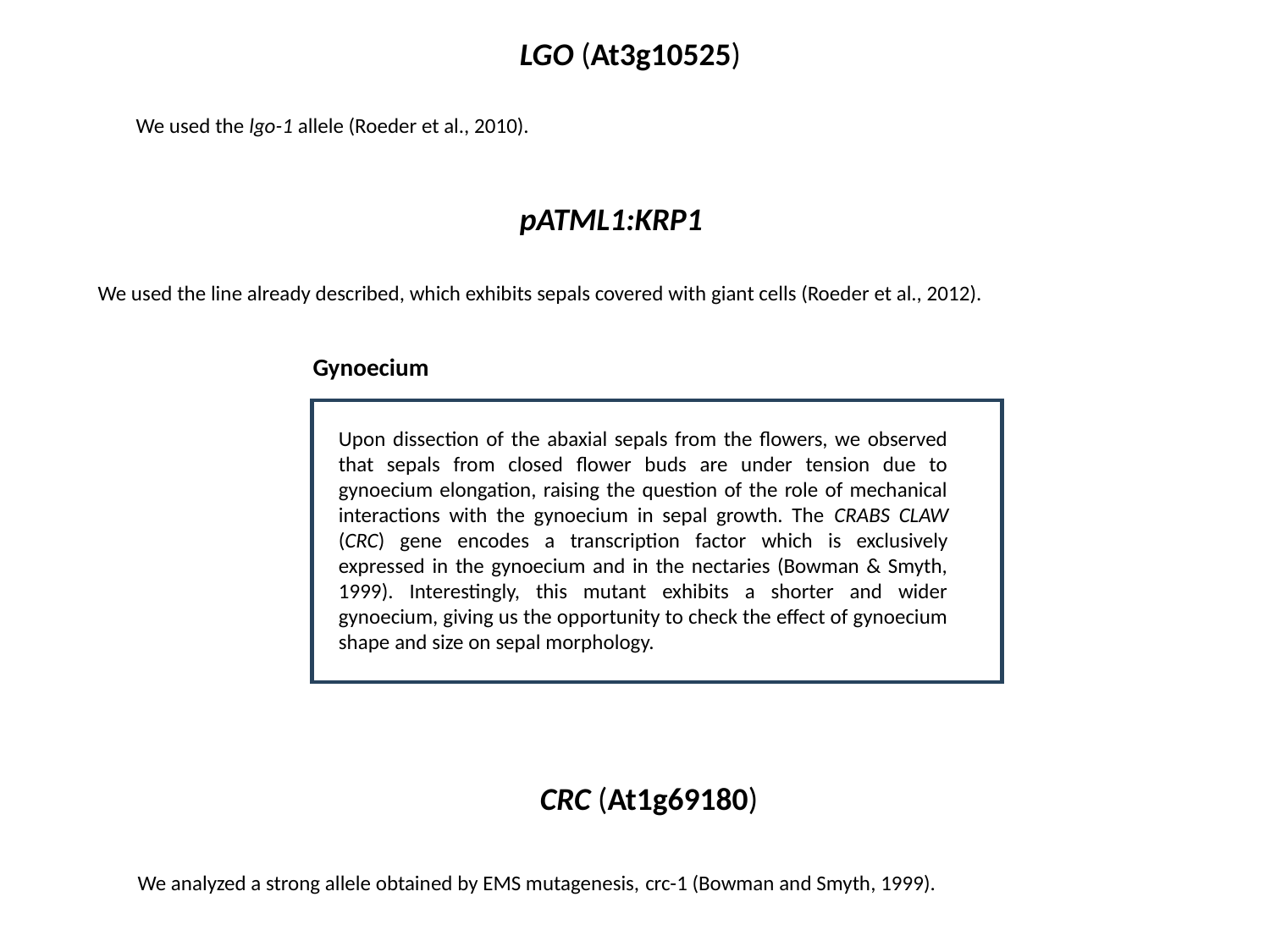

LGO (At3g10525)
We used the lgo-1 allele (Roeder et al., 2010).
pATML1:KRP1
We used the line already described, which exhibits sepals covered with giant cells (Roeder et al., 2012).
Gynoecium
Upon dissection of the abaxial sepals from the flowers, we observed that sepals from closed flower buds are under tension due to gynoecium elongation, raising the question of the role of mechanical interactions with the gynoecium in sepal growth. The CRABS CLAW (CRC) gene encodes a transcription factor which is exclusively expressed in the gynoecium and in the nectaries (Bowman & Smyth, 1999). Interestingly, this mutant exhibits a shorter and wider gynoecium, giving us the opportunity to check the effect of gynoecium shape and size on sepal morphology.
CRC (At1g69180)
We analyzed a strong allele obtained by EMS mutagenesis, crc-1 (Bowman and Smyth, 1999).
